# Differential host mortality explains the effect of high temperature on the prevalence of a marine pathogen

**DOI:** 10.1371/journal.pone.0187128

**Published:** 2017-10-30

**Authors:** Timothy J. Sullivan, Joseph E. Neigel

**Affiliations:** Department of Biology, the University of Louisiana at Lafayette, Lafayette, Louisiana, United States of America; University of New South Wales, AUSTRALIA

## Abstract

Infectious diseases threaten marine populations, and the extent of their impacts is often assessed by prevalence of infection (the proportion of infected individuals). Changes in prevalence are often attributed to altered rates of transmission, although the rates of birth, recovery, and mortality also determine prevalence. The parasitic dinoflagellate *Hematodinium perezi* causes a severe, often fatal disease in blue crabs. It has been speculated that decreases in prevalence associated with high temperatures result from lower rates of infection. We used field collections, environmental sensor data, and high-temperature exposure experiments to investigate the factors that change prevalence of infections in blue crab megalopae (post-larvae). These megalopae migrate from offshore waters, where temperatures are moderate, to marshes where temperatures may be extremely high. Within a few days of arriving in the marsh, the megalopae metamorphose into juvenile crabs. We found a strong negative association between prevalence of *Hematodinium* infection in megalopae and the cumulative time water temperatures in the marsh exceeded 34°C over the preceding two days. Temperatures this high are known to be lethal for blue crabs, suggesting that higher mortality of infected megalopae could be the cause of reduced prevalence. Experimental exposure of megalopae from the marsh to a temperature of 34°C resulted in higher mortality for infected than uninfected individuals, and decreased the prevalence of infection among survivors from 18% to 3%.

## Introduction

**Fig 1:**
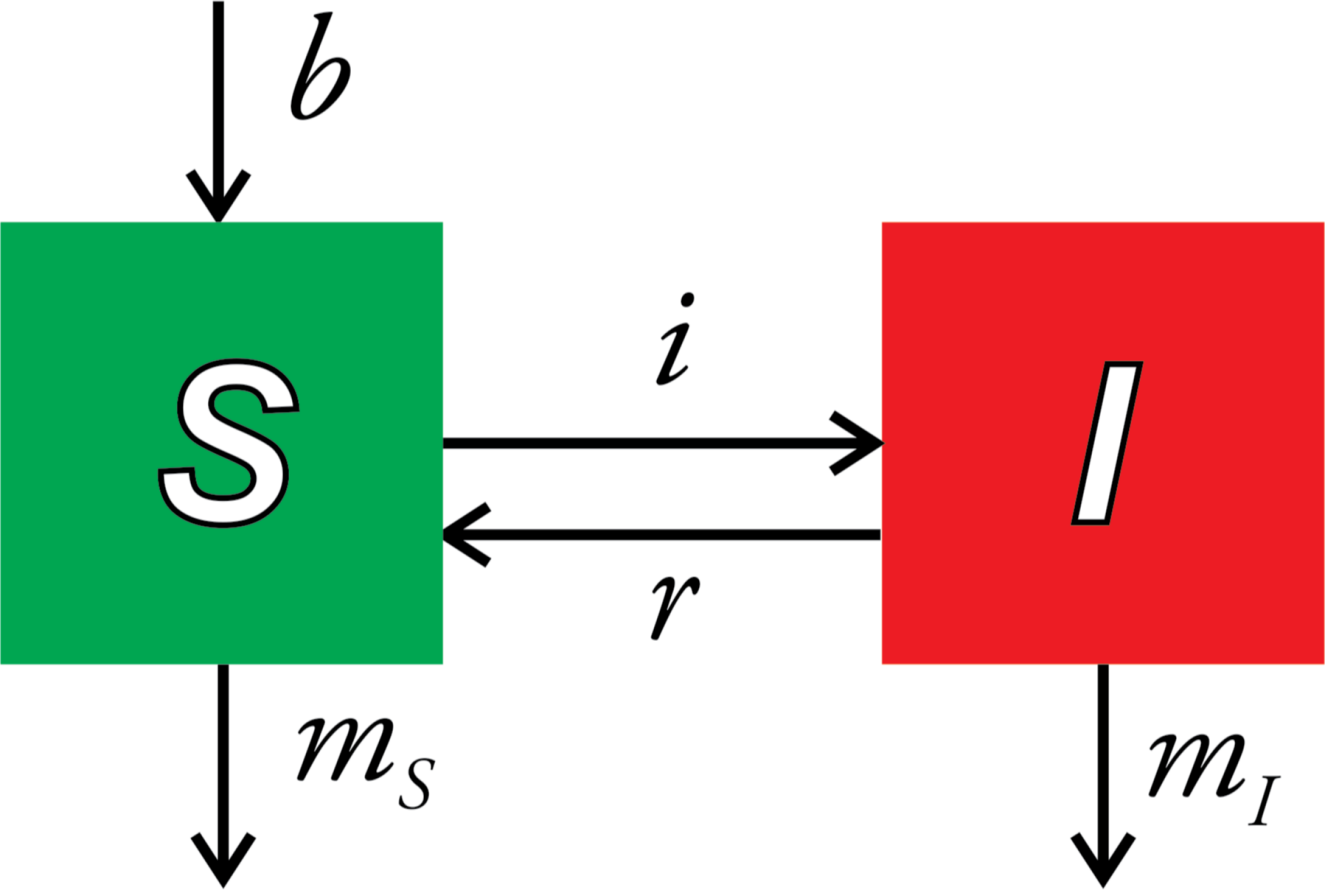
*SI* model of prevalence. Boxes represent groups that differ in disease status, with numbers of *S* susceptible and *I* infected individuals. Prevalence is the fraction of infected individuals *I*/(*I*+*S*). Arrows represent transitions with rates for birth (*b*), infection (*i*), recovery (*r*), and mortality (*m*).

Outbreaks of infectious diseases in marine animals are of growing concern. Pollution, non-native pathogens, and climate change are generally expected to increase the frequency and severity of marine disease outbreaks and their effects on ecosystems, fisheries, and threatened species [1–4], although some research has suggested these effects will be taxon-specific [5, 6]. Models that accurately predict outbreaks are needed to develop strategies for limiting their impacts [7–9]. However, well-developed epidemiological models for human and wildlife populations are not easily applied to marine systems [1, 3, 10]. The parameters of these models represent processes that are difficult to observe or quantify in the marine realm, such as disease transmission, disease-induced mortality, and recovery from infection [2, 3, 10]. In some cases, basic etiological aspects of a disease, such as the identity of the pathogen or its mode of transmission, are unknown [3, 11–13]. Consequently, efforts to identify the causes of marine outbreaks and predict their occurrences have focused on finding associations between environmental factors, such as temperature and salinity, and disease prevalence, the proportion of infected or diseased individuals in a population [14–20]. However, the use of prevalence, as an indicator of the progress or severity of an outbreak is potentially misleading. The rates of multiple processes, including infection, recovery from infection, birth of new individuals, and the deaths of both infected and uninfected individuals jointly determine disease prevalence [Fig 1, 21, 22]. Environmental factors could influence any of these processes, so that correlations between environmental variables and disease prevalence can arise in different ways with different implications for host populations.

The parasitic dinoflagellate *Hematodinium perezi* is a widespread and virulent pathogen of the blue crab, *Callinectes sapidus*. In laboratory experiments, mortality of infected adult blue crabs has ranged from 87 to 100% [23]. Prevalence of infection along the US Atlantic coast is often as high as 30% and may approach 100% in severe outbreaks [24]. Prevalence of infection by *H*. *perezi* in blue crabs is correlated with both water temperature and salinity [14, 24]. Infected blue crabs are rarely found at salinities below 18 ppt and have not been reported at salinities below 11 ppt [15, 24]. There is evidence that low salinity reduces prevalence by reducing the rate of infection. The free-living dinospore stage of *H*. *perezi*, which may be responsible for transmission of disease, does not tolerate low salinities [25, 26]. However, exposure of an infected host to low salinity does not eliminate the infection [25]. Prevalence also is associated with temperature, although the relationship is more complex. Prevalence is generally highest during the fall or early winter when water temperatures are relatively low, and may decline in late winter when temperatures are lowest [14, 15, 24]. Prevalence increases in spring as water temperatures rise, but later declines in summer when temperatures are highest [14, 24], although this pattern is not always followed [27]. Prior to the results of that study [27], it has been suggested that extreme temperatures could inhibit either transmission or establishment of infection, with optimum temperatures for the pathogen in the moderate range of 15 to 18°C [14, 24].

**Fig 2:**
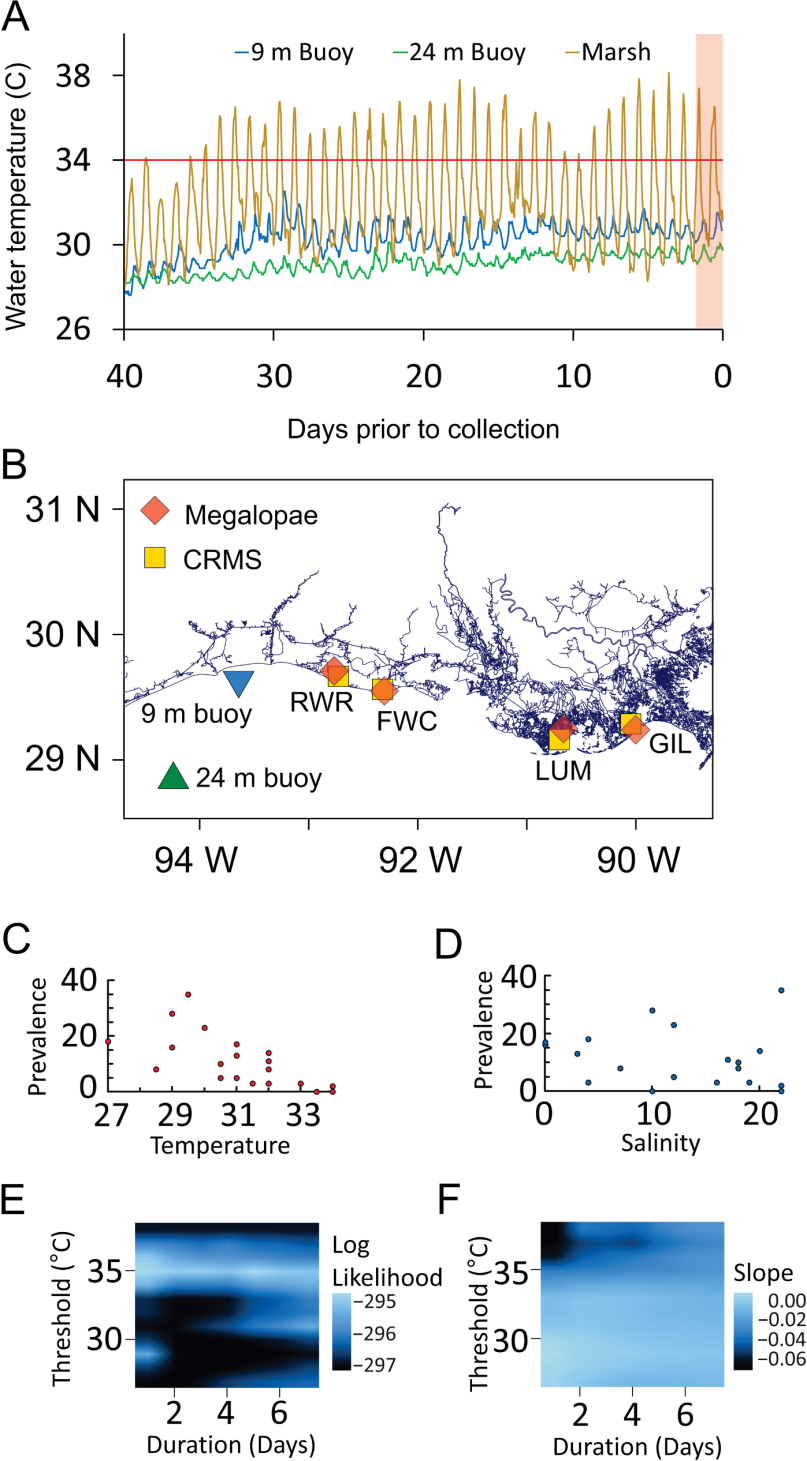
Effect of temperature and salinity on prevalence of *H*. *perezi* in blue crab megalopae. (*A*) Hourly water temperature at Coastwide Reference Monitoring System (CRMS) station 0581 near the RWR marsh site (yellow), nearshore buoy 42051 (blue) and offshore buoy 42050 (green) for 40 days prior to collection of megalopae at RWR on Aug. 11, 2015. Red line indicates high-temperature threshold of 35°C, red-shaded area indicates 2-day period prior to megalopae collection. (*B*) Megalopae were collected from marshes at Rockefeller Wildlife Refuge (RWR), Freshwater City Locks (FWC), Louisiana Universities Marine Consortium (LUM), and Grand Isle Marine Laboratory (GIL). Temperature and salinity data from CRMS sites 0178 (at GIL), 0347 (at LUM), 0581 (at RWR) and 0633 (at FWC). Temperature data from buoys at stations 42050 (TABS F; green) and 42051 (TABS R; blue). (*C*) Prevalence, as percentage of megalopae from which a portion of the *H*. *perezi* 18S rRNA gene was PCR-amplified, plotted against temperature (°C) at collection time and location. (*D*) Prevalence plotted against salinity (ppt) at collection time and location. (*E*) Log-likelihoods and (*F*) slopes from logistic regression models that predict log odds ratio of infection from proportion of hourly marsh temperatures that exceeded each threshold, with variable threshold temperatures and time intervals. Increasing log-likelihood values (lighter color) indicate models with combinations of threshold temperature and time intervals with greater support. Steeper downward slopes (darker colors) indicate models in which thermal stress has a larger negative effect on prevalence of infection.

Juvenile and adult blue crabs from the low-salinity marshes of Louisiana are generally not infected by *H*. *perezi*. However, *H*. *perezi* has recently been detected in adult blue crabs from high-salinity shoals off the coast and in blue crab megalopae settling in low-salinity marshes of Louisiana [28]. This pattern of occurrence is consistent with the life-cycle of the blue crab, which involves migrations between habitats that differ greatly in temperature and salinity. After reaching maturity and mating in the marsh, females migrate offshore to spawn [29]. Larvae of the blue crab are planktonic for 5–6 weeks in high-salinity offshore waters before entering the post-larval megalopal stage and moving to low-salinity marshes where they metamorphose into juveniles within ~1–2 days [30, 31]. In contrast to the constant and moderate conditions of offshore waters where blue crab larvae develop, summer water temperatures in the marsh vary considerably among locations, change rapidly, and often reach extreme highs (Fig 2A and S1 Fig). The brief period between the arrival of megalopae in the marsh and their metamorphosis into juveniles provides an opportunity to investigate the short-term effects of local environmental conditions on the prevalence of *H*. *perezi*.

## Materials and methods

### Calculation of proportion of hours above high temperature threshold

Hourly recordings of temperature and salinity were downloaded from the Louisiana Coastwide Reference Monitoring System (CRMS) database (www.lacoast.gov/crms) for CRMS sites near each of the four sampling locations (Fig 2B; S1 Table). The precision of CRMS temperature and salinity measurements are 0.1°C and 0.1 ppt, respectively. CRMS sites were between 2 and 10 km from the sampling locations (Fig 2B; S1 Table) and within the same estuarine-marsh systems. Temperatures recorded at sampling locations were usually within 1–2°C of temperatures simultaneously recorded at the nearest CRMS site. A Perl script was used to parse CRMS data and calculate the percentage of hourly recordings, over a designated time interval, that exceeded a designated threshold temperature.

### Megalopae collection

Blue crab megalopae were sampled from marshes bordering four Louisiana estuaries (Fig 2B): Grand Isle (GIL), Louisiana Universities Marine Consortium (LUMCON; LUM), Freshwater City (FWC), and Rockefeller Wildlife Refuge (RWR) in 2010, 2013, 2014, 2015, and 2016 (S1 Fig; S1 Table) with access from the Louisiana Department of Wildlife and Fisheries, the Louisiana Universities Marine Consortium, and the U.S. Army Corps Of Engineers. Between 5 and 8 passive “hogs-hair” (air conditioning filter) collectors (S2 Fig)[32] were deployed for ~24 hours at each location. The collectors were removed the following day and rinsed with ambient seawater. Immediately after passive collectors were removed, a 253 μm plankton net (Wildco) was towed over a distance of ~200 meters at the same location. Megalopae from both passive collectors and plankton nets were washed on a 200 μm filter with ambient seawater and all *Callinectes sapidus* megalopae were individually removed while still alive. The megalopae were washed a second time with ambient seawater before being preserved whole in pre-chilled 95% ethanol at 4°C. Measurements of salinity and water temperature with precisions of 1 ppt and 0.5°C respectively were taken at the time of each collection.

### Extraction of DNA and detection of *Hematodinium perezi* by PCR

DNA was extracted from whole ethanol-preserved megalopae using NucleoSpin® 96 Tissue Kits (Machery-Nagel) and an epMotion 5075 TMX liquid handling workstation (Eppendorf) following the manufacturer’s protocol. The presence of DNA of *H*. *perezi* was detected by PCR amplification of a portion of the 18S ribosomal RNA gene using the primers Hemat-F-18S and Hemat-R-18S developed by Friedman and co-workers [33]. DNA concentrations were determined with a Nanodrop spectrophotometer (Thermo Scientific). PCR reactions were in 15 μl with 1X AmpliTaq Gold^®^ PCR Buffer (Applied Biosystems), 2.5mM MgCl_2_, 1 mM dNTPs, 1.2 μM of each forward and reverse primer, 0.6 units of AmpliTaq^®^ Gold (Applied Biosystems), 20 ng of DNA, and Milli-Q^®^ water. The amplification profile was 95°C for 5 minutes, then 40 cycles of: 96°C for 15 seconds, 56°C for 30 seconds 72°C for 45 seconds, and lastly 72°C degrees for 10 minutes. Positive and negative controls were included in all reactions. PCR products were electrophoresed on 2% agarose gels with 0.05% ethidium bromide and visualized on a Molecular Imager ® Gel Doc ™ XR system (Bio Rad).

### Modelling associations of prevalence with physical and environmental variables

Effects of environmental variables on the probability of infection (and consequently prevalence) of *H*. *perezi* were investigated with logistic regressions in R 3.2.0. For our initial model, we used salinity and temperature measurements taken at the time and location of collection. In this model, both temperature and salinity were significantly associated with *H*. *perezi* prevalence, with higher salinity increasing prevalence and higher temperature decreasing prevalence (S2 Table). In our second model, we used variables that were intended to represent the amount of time that megalopae were exposed to stressful extremes of temperature or salinity. The thermal stress variable was the proportion of hourly CRMS measurements above 35°C for the 2 days (48 hours) prior to collection, the salinity stress variable was the proportion of hourly CRMS measurements above 11 ppt for 2 days prior to collection. In this model, greater thermal stress significantly decreased prevalence while greater salinity stress had no significant effect (S2 Table). Prior to running these models, we tested multicollinearity between the predictor variables using the vif command in the *cars* package of R 3.2.0. A vif value less than 4 was interpreted as absence of collinearity.

Additional models were used to determine if there was statistical support for a distinct threshold temperature for the effect of thermal stress on prevalence of infection, and if so, did it correspond to the threshold of 35°C and the time span of 48 hours that we had predicted. Model likelihoods were compared for a series of logistic regression models that predicted the log of the odds ratio of infection (probability of being infected divided by probability of not being infected) from the thermal stress variable (proportion of preceding hourly CRMS measurements above a high-temperature threshold). Each model used a different combination of the duration parameter (the time span over which the proportion of hours was calculated) and the threshold parameter (the temperature threshold for thermal stress). The durations used in these models ranged from 1 to 7 days in increments of 1 day and the thresholds ranged from 27°C to 39°C in 1°C increments, for a total of 91 models. We plotted the log-likelihood and slope (log of the infection odds ratio vs. the heat stress predictor variable) from each model on linear interpolated surface plots using the *ggplot2* package in R 3.2.0. For the plot of log-likelihoods, lighter colors indicate models with higher likelihoods, and thus greater statistical support. For the plot of slopes, darker colors represent steeper downward slopes where increases in thermal stress have larger negative effects on prevalence of infection.

### Thermal stress experimental manipulation

On the morning of May 15, 2016 we collected megalopae of *C*. *sapidus* from the dock of LUMCON (Chauvin, LA) on “hogs-hair” collectors. Six groups of 20 megalopae were divided among three control and three experimental chambers. All chambers contained 2 L of seawater and 10 cm^2^ of “hogs-hair” air conditioner filter. Both control and treatment chambers were maintained at ambient salinity conditions (4 ppt). The control chambers were maintained at 27°C, the water temperature at the time of collection. In the treatment chambers, individuals were acclimated for 2 hours at 27°C and then the temperature was increased by 1°C every two hours for 14 hours and then held at 34°C for 24 hours. We chose 34°C because our field results showed that prevalence decreased with temperature, reaching zero at 34°C. A 1-day (24 hour) exposure was chosen to correspond to the average length of time megalopae would have been in the marsh at the time of collection (assuming 2 days between arrival and metamorphosis), and because our thermal stress models showed greatest highest likelihoods for durations of 1–2 days. The concentrations of ammonia and pH were measured using water testing kits (Aquarium Pharmaceuticals) before and after the experiment (S3 Table). Light conditions in the room where the experiment was conducted followed the local photoperiod. Megalopae were fed a mixture of nauplii of *Artmeia* sp. and copepods every 10 hours. Once the trial was completed, megalopae were sorted into live and dead individuals and preserved in pre-chilled 95% ethanol at 4°C. Following the experiment, DNA was extracted from each individual megalopa and individually screened for the presence of *H*. *perezi* following the protocol outlined above. Significance of differences in prevalence and mortality for control and treatment and for infected and uninfected groups were compared using Cochran-Mantel-Haentzel tests in R. 3.2.0.

## Results

We collected settling megalopae from four locations on the coast of Louisiana between 2010 and 2016 (Fig 2B). Prevalence, defined here as the percentage of megalopae testing positive in a PCR assay for *H*. *perezi* DNA [28], ranged from 0 to 35% (S1 Table). Water temperature and salinity were measured at the time and location of collection and at hourly intervals by nearby CRMS monitoring stations [34]. We used logistic regression to explore the effects of environmental factors on *H*. *perezi* prevalence. Both salinity and temperature at the point of collection had significant effects on prevalence (Fig 2C and 2D, S2 Table), with prevalence generally increasing with salinity and decreasing with temperature.

We used additional models to investigate the possibility of a threshold for the effect of high temperature on prevalence by varying both the threshold temperature and the duration of the preceding time interval over which the proportion of hourly temperature measurements above the threshold was calculated. A threshold of 35°C maximized model likelihood values for all durations (Fig 2E). At 34°C the slope of the relationship between prevalence and threshold temperature changed sharply from near zero to negative. Above 34°C, higher threshold temperatures decreased prevalence, most noticeably for durations of 1–2 days (Fig 2F). The 1–2 day time interval is consistent with an effect of the local environment on megalopae between their arrival in the marsh and their metamorphoses into juveniles within 2 days. The threshold of 34–35°C suggests a stressful or lethal effect on the host because previous work has shown significant mortality occurs in juvenile blue crabs following short-term exposures to temperatures above 35°C [35]. Based on these observations, we considered two hypotheses for the negative effect of high temperatures on prevalence of *H*. *perezi* infection in blue crab megalopae. The first hypothesis, the “heat-cure hypothesis”, is that stressfully high temperatures result in rapid elimination of *H*. *perezi* from the host. Examples of a lethal effect of high temperature on pathogens include the killing of eggs of the monogenean *Heterobothrium okamotoi* and cysts of the ciliate *Cryptocaryon irritans* [36]. High temperature also has been shown to eliminate the toxic dinoflagellates *Gymnodinium catenatum* and *Alexandrium catenell*, from ballast water [37]. Finally, exposure of reef building corals to high temperature results in the rapid expulsion of their symbiotic dinoflagellates [38]. The second hypothesis, the “heat-kill hypothesis”, is that high temperatures kill infected megalopae at a higher rate than uninfected megalopae.

**Fig 3:**
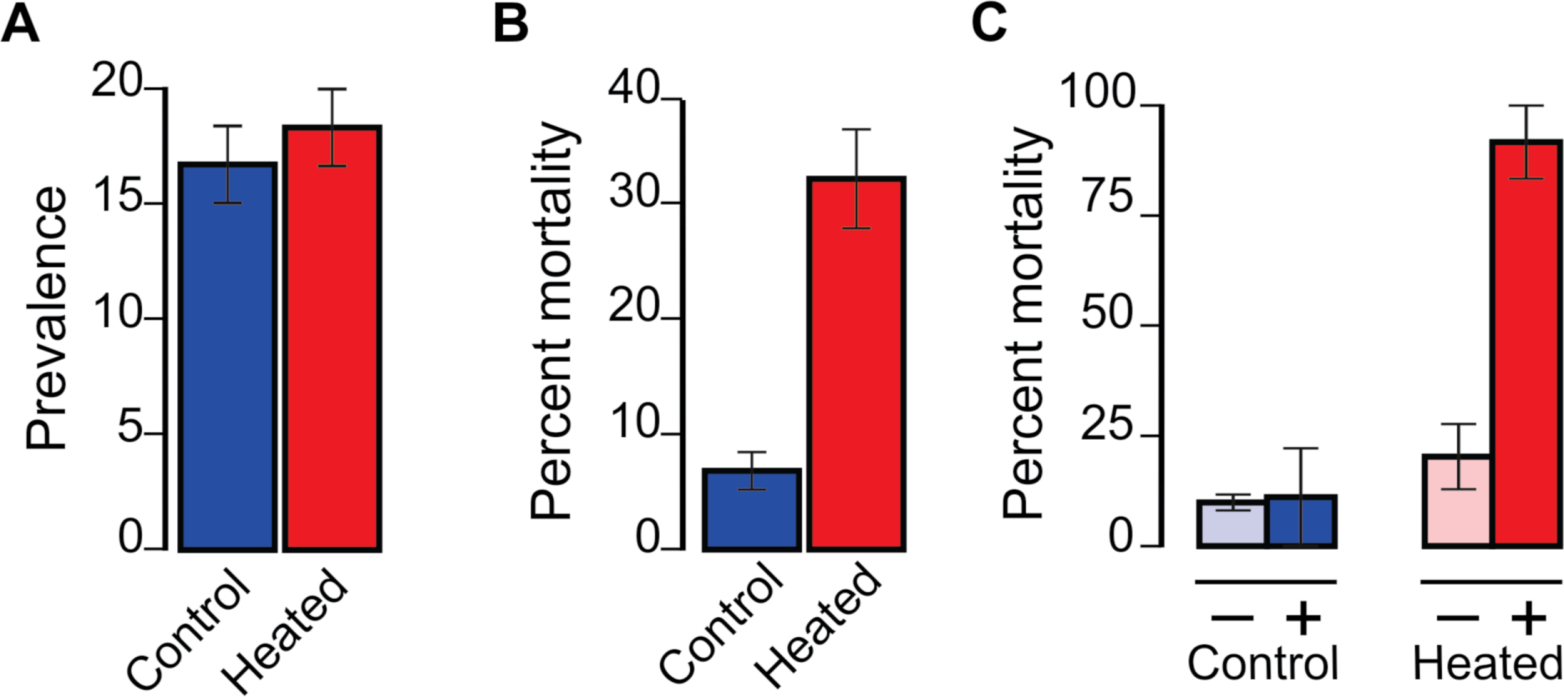
Heat treatment experiment. (*A*) Total (live + dead) prevalence of *H*. *perezi* compared between control (blue) and heat-treatment (red) groups (*χ*^2^ = 0.06, d.f. = 1, *P* = 0.81). (*B*) Overall percent mortality of megalopae compared between control (blue) and heat-treatment (red) groups (*χ*^2^ = 13.10, d.f. = 1, *P* = 0.0003). (*C*) Percent mortality compared between infected (+) and uninfected (**-**) megalopae in control (blue) groups (*χ*^2^ = 0.23, d.f. = 1, *P* = 0.63) and heat-treatment (red) groups (*χ*^2^ = 19.7, d.f. = 1, *P* < 0.00001). Bar graphs show mean values for control and treatment tanks (3 tanks for each group with 20 individuals per tank). Error bars represent standard error. For raw data see S4 Table.

We performed an experiment to test the heat-cure and heat-kill hypotheses. Megalopae, some of which were anticipated to be infected with *H*. *perezi*, were collected on artificial substrates suspended in the water column at the field station of the Louisiana Universities Marine Consortium (LUMCON). This method collects only living, actively settling megalopae. After collection, groups of 20 megalopae (3 treatment groups and 3 control groups) were either treated with an increase in temperature to a high of 34°C (HT) or maintained at the ambient water temperature of 27°C as controls. Megalopae in the treatment groups (11 individuals infected of 60, Prevalence = 18%) did not differ significantly from the control groups (10 individuals infected of 60, Prevalence = 17%) in the proportions of individuals (living and dead) in which *H*. *perezi* was detected (Fig 3A; *χ*^2^ = 0.06, d.f. = 1, *P* = 0.81), providing no support for the heat-cure hypothesis. The overall mortality rate for megalopae exposed to the HT treatment (20 dead individuals of 60, Mortality = 33%) was higher than the rate for controls (4 individuals dead of 60, Mortality = 7%), confirming that 34°C is stressful for blue crab megalopae (Fig 3C; *χ*^2^ = 13.10, d.f. = 1, *P* = 0.0003). In the HT treatment, the mortality rate for infected megalopae (10 individuals of 11, Mortality = 92%) was 4.5 times greater than for uninfected megalopae (10 individuals of 49, Mortality– 20%, *χ*^2^ = 19.7, d.f. = 1, *P* < 0.00001), whereas in control groups the mortality rates for infected (1 individual of 10, Mortality = 10%) and uninfected individuals (3 individuals of 50, Mortality = 7%) were low and not significantly different (Fig 3D; *χ*^2^ = 0.23, d.f. = 1, *P* = 0.63). This effect of HT corresponds to a change in prevalence from 18% to 3% among surviving megalopae.

The infection status of individual megalopae was not directly manipulated in this experiment, and so susceptibility to infection and sensitivity to heat stress could have both been caused by some additional, unobserved factor. Nevertheless, mortality was greater for infected individuals, supporting the heat-kill hypothesis. Differential mortality after exposure to high temperatures for less than 2 days can thus explain the association between high temperatures and lower prevalence of infection by *H*. *perezi* among blue crab megalopae settling in the marsh.

## Discussion

Our results revealed a strong negative relationship between high environmental temperatures and the prevalence of infection of blue crab megalopae by *H*. *perezi*, which appears to be driven by higher mortality of infected individuals. These findings raise the question of whether changes in mortality rates also contribute to the effects of environmental factors on the prevalence of *H*. *perezi* infection in juvenile and adult blue crabs. High rates of morality among infected blue crabs in the laboratory (up to 100%) suggest this mechanism could have large effects and should be evaluated. Decreases in the prevalence of *H*. *perezi* infection of blue crabs in the field are associated with both low and high extremes of water temperature [14, 15, 24] that are consistent with temperatures known to be stressful for adult blue crabs [35, 39, 40]. In short-term laboratory experiments with uninfected individuals, stress occurs at temperatures below 13°C or above 30°C and significant mortality occurs below 5°C or above 33°C [35, 39, 40]. At present, no other mechanism has been demonstrated for an effect of temperature on the prevalence of *H*. *perezi* in adult blue crabs, although it has been reported that growth of *H*. *perezi in vitro* is inhibited at 10°C [41].

Previous investigations of associations between environmental factors and the prevalence of infection in blue crabs by *H*. *perezi* have considered changes in the rate of infection as the hypothetical underlying mechanism [14], though a recent study of rates of infection of blue crabs by *H*. *perezi* found mortality may be more important than generally assumed [26]. Our results and those of Huchin-Mian and colleagues [26] are significant in that associations mediated by changes in mortality rates would have very different implications for the host population. A decrease in prevalence that might be viewed as a sign that an outbreak was slowing down might instead be signaling an increase in mortality, especially when considering the potential effects of rapid environmental forcing on the dynamics of infection ([26] and this study). However, even if it were established that changes in mortality rates cause changes in prevalence, it would be difficult to gauge their demographic consequences without an understanding of the pathogen’s effects on the host’s entire life cycle. For example, although we have shown that high temperatures in the marsh are likely to increase mortality among infected megalopae, we do not know if this would lead to decreased recruitment to the juvenile stage. Although low settlement rates have been linked to low rates of recruitment in blue crab populations on the Atlantic coast, settlement rates in the northern Gulf of Mexico are much higher and may not be limiting [42]. Furthermore, even without heat stress, infected megalopae may nevertheless die after settling, with the same effect on recruitment. That would explain the apparent absence of infected juveniles or adults in the low-salinity marshes of Louisiana [28].

The disease caused by *H*. *perezi* in blue crabs is one of the most thoroughly-studied marine invertebrate diseases, but there remain critical gaps in our understanding of how the pathogen is transmitted, how it affects each life stage, and the causes of associations between environmental variables and disease prevalence. Monitoring of susceptible populations is needed to better predict, manage, and prevent marine disease outbreaks in the face of emerging pathogens, climate change and other challenges [1, 7–9]. For disease monitoring, prevalence is a simple, familiar, and frequently used measure. However, as Anderson and May showed in their seminal paper on the population biology of infectious diseases [21], demographic processes such as disease-induced mortality that may be irrelevant in epidemiological models of human populations can be important in the dynamics of natural populations. In species with multiphasic life histories, such as the blue crab, the relationship between prevalence and the epidemiological processes that determine prevalence may be too complex to permit simple assessment of the impact of the disease on the host population. In such cases, combining field observations with experimental data can help reveal critical aspects of these complex disease mechanisms. This integrated approach, and a focus on not only larvae but surveys of the entire host life cycle should be included in future studies of *H*. *perezi* and other marine diseases, as it may provide crucial information for disease monitoring, prediction, and hopefully mitigation efforts.

## Supporting information

S1 Fig Water temperatures experienced offshore by larvae and in marsh by megalopae from all collections. Hourly water temperature at CRMS stations near each marsh site (yellow), nearshore buoy 42051 (blue) and offshore buoy 42050 (green) for 40 days prior to collection of megalopae. Red line indicates high-temperature threshold.
(PDF)

S2 Fig Photograph showing a hogs-hair collector for the passive collection of settling blue crab megalopae. (PDF)

S1 Table Prevalence of *Hematodinium* detected in megalopae collected in Louisiana *estuaries*. (PDF)

S2 Table *H. perezi* model results. Model results for logistic regressions evaluating *H*. *perezi* prevalence and both (**A**) point estimates of temperature and salinity and (**B**) derived proportional measurements of lethal temperature stress and the time salinity was above 11 ppt.
(PDF)

S3 Table Conditions from control and heat-treatment chambers before and after 40 h experiment. (PDF)

S4 Table Mortality and prevalence results from the heat treatment exposure experiment including both count and percentage data. (PDF)
