# Supplementary material for: Differential host mortality explains the effect of high temperature on the prevalence of a marine pathogen
Source: PLoS One. 2017 Oct 30;12(10):e0187128. doi: 10.1371/journal.pone.0187128 (PMC5662175; doi:10.1371/journal.pone.0187128)

S1 Fig. Water temperatures experienced offshore by larvae and in marsh by megalopae from all collections. Hourly water temperature at CRMS stations near each marsh site (yellow), nearshore buoy 42051 (blue) and offshore buoy 42050 (green) for 40 days prior to collection of megalopae. Red line indicates high-temperature threshold.

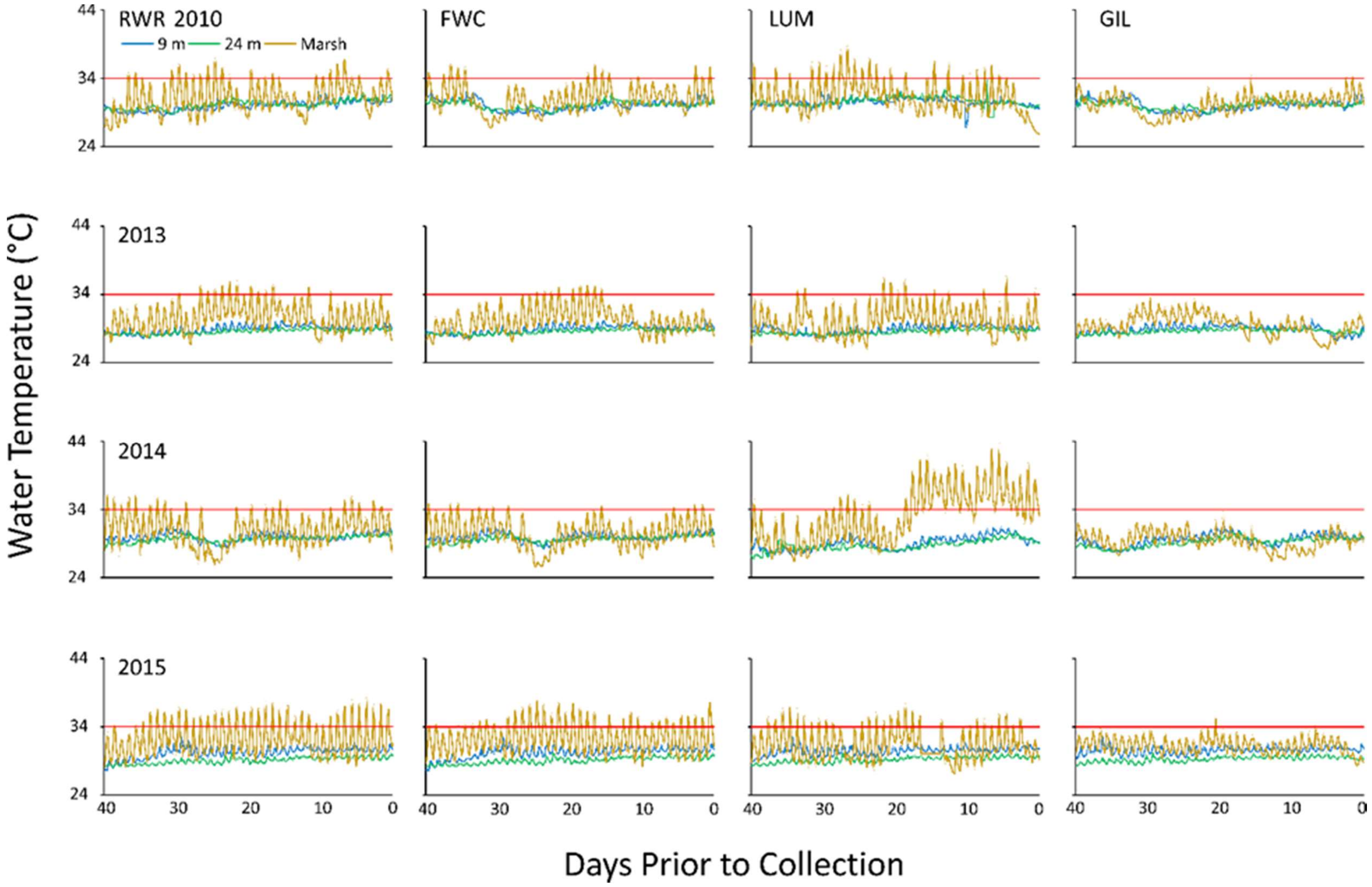

Supplement: S1 Fig — Hourly water temperature at CRMS stations near each marsh site (yellow), nearshore buoy 42051 (blue) and offshore buoy 42050 (green) for 40 days prior to collection of megalopae. Red line indicates high-temperature threshold. (PDF) [file pone.0187128.s001.pdf]
