# Supplementary figures and images for: Differential host mortality explains the effect of high temperature on the prevalence of a marine pathogen
Source: PLoS One. 2017 Oct 30;12(10):e0187128. doi: 10.1371/journal.pone.0187128 (PMC5662175; doi:10.1371/journal.pone.0187128)

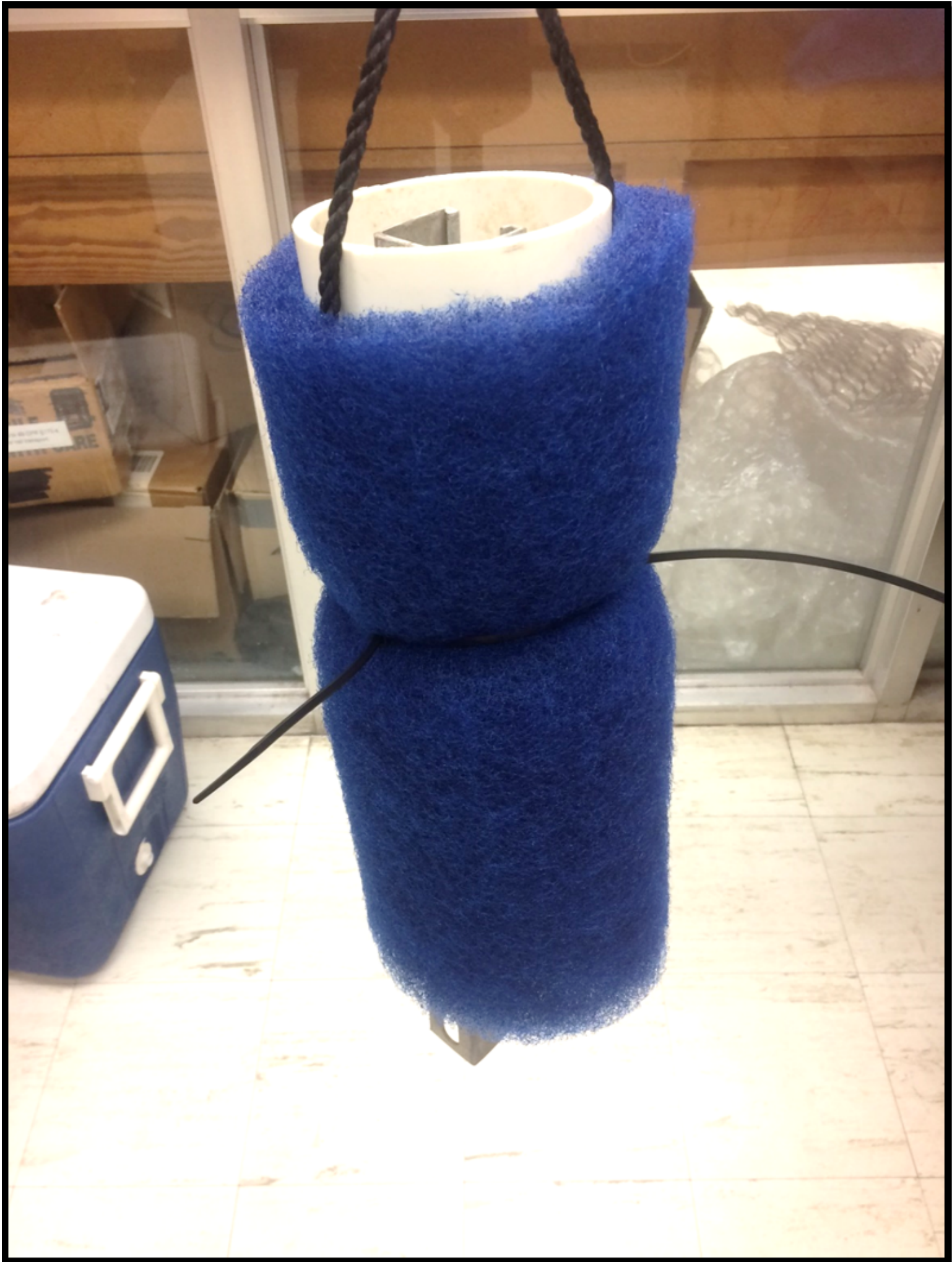

Supplement: S2 Fig — (PDF) [file pone.0187128.s002.pdf]
