# Supplementary material for: Differential host mortality explains the effect of high temperature on the prevalence of a marine pathogen
Source: PLoS One. 2017 Oct 30;12(10):e0187128. doi: 10.1371/journal.pone.0187128 (PMC5662175; doi:10.1371/journal.pone.0187128)
Supplement: S1 Table — (PDF) [file pone.0187128.s003.pdf]

S1 Table. Prevalence of *Hematodinium* detected in megalopae collected in Louisiana estuaries.

Location	Latitude	Longitude	CRMS	Date	Salinity	Temperature	N Total	N Infected	Prevalence
GIL	29.24	90.00	0178	7/31/2010	12	30	35	8	23
				8/31/2013	18	32	38	3	8
				8/01/2014	22	29.5	43	15	35
				8/18/2015	12	30.5	40	2	5
				7/26/2016	12	31	55	3	5
				Total			211	31	15
LUM	29.25	90.66	0347	8/29/2010	4	33	36	1	3
				8/21/2013	18	30.5	39	4	10
				7/18/2014	7	28.5	37	3	8
				8/16/2015	10	33.5	40	0	0
				5/15/2016	4	27	120	21	18
				Total			272	29	11
FWC	29.55	92.31	0633	8/01/2010	20	32	36	5	14
				8/25/2013	22	34	45	1	2
				8/12/2014	10	29	39	11	28
				8/11/2015	19	31.5	32	1	3
				7/06/2016	3	31	32	4	13
				Total			184	22	12
RWR	29.71	92.77	0581	8/09/2010	17	32	36	4	11
				8/25/2013	22	34	13	0	0
				8/12/2014	0	29	38	6	16
				8/11/2015	16	32	35	1	3
				7/06/2016	0	31	24	4	17
				Total			146	15	10
	Totals from all collections						813	97	12
