# Supplementary material for: Differential host mortality explains the effect of high temperature on the prevalence of a marine pathogen
Source: PLoS One. 2017 Oct 30;12(10):e0187128. doi: 10.1371/journal.pone.0187128 (PMC5662175; doi:10.1371/journal.pone.0187128)
Supplement: S2 Table — Model results for logistic regressions evaluating H. perezi prevalence and both (A) point estimates of temperature and salinity and (B) derived proportional measurements of lethal temperature stress and the time salinity was above 11 ppt. (PDF) [file pone.0187128.s004.pdf]

S2 Table. *H. perezi* model results. Model results for logistic regressions evaluating *H. perezi* prevalence and both (**A**) point estimates of temperature and salinity and (**B**) derived proportional measurements of lethal temperature stress and the time salinity was above 11 ppt.

Model		Coefficients	Estimate	Std. Error	*Z*	*P*	Odds
(A)	Point Estimate	Intercept	8.66	1.99	4.4	<0.0001	5768
		Salinity	0.05	0.02	2.5	0.013	1.05
		Temperature	-0.37	0.07	-5.2	<0.0001	0.69
(B)	Derived Real-time	Intercept	-1.6	0.19	-8.5	<0.0001	0.19
		Day2_Temp35	-0.04	0.02	-2.02	0.044	0.97
		Day2_Salt11	-0.003	0.002	-1.3	0.19	0.99
