# Supplementary material for: Differential host mortality explains the effect of high temperature on the prevalence of a marine pathogen
Source: PLoS One. 2017 Oct 30;12(10):e0187128. doi: 10.1371/journal.pone.0187128 (PMC5662175; doi:10.1371/journal.pone.0187128)
Supplement: S3 Table — (PDF) [file pone.0187128.s005.pdf]

S3 Table. Conditions from control and heat-treatment chambers before and after 40 h experiment.

	Before Experiment				After 40 hours			
Group	Temperature (˚C)	Salinity	pH	Ammonia (ppm)	Temperature (˚C)	Salinity	pH	Ammonia (ppm)
Control 1	27	4	8	0	27	4	8	0-0.25
Control 2	27	4	8.2	0.25	27	4	8.2	0.25-0.5
Control 3	27	4	8	0	27	4	8.2	0.25
Heated 1	27	4	8.2	0.25	34	4	8.2	0.25-0.5
Heated 2	27	4	8.2	0	34	4	8.2	0.25
Heated 3	27	4	8	0	34	4	8	0.25
