# Supplementary material for: Differential host mortality explains the effect of high temperature on the prevalence of a marine pathogen
Source: PLoS One. 2017 Oct 30;12(10):e0187128. doi: 10.1371/journal.pone.0187128 (PMC5662175; doi:10.1371/journal.pone.0187128)
Supplement: S4 Table — (PDF) [file pone.0187128.s006.pdf]

Supplementary Table S4: Infection and high temperature exposure increase mortality of blue crab larvae. *N* = number of individuals, Mortality = number (Count) or percent (%) of dead individuals, Prevalence = number (count) or Percent % of individuals that were infected by *H. perezi*.

Group	Tank	*N*	Prevalence (%)	Prevalence (Count)	Mortality (%)	Mortality (Count)	Prevalence (Live %)	Prevalence (Dead %)	Infected Mortality (%)	Uninfected Mortality (%)
Control	1	20	20	4	5	1	21	0	0	6.3
Control	2	20	15	3	10	2	17	0	0	12
Control	3	20	15	3	5	1	11	33	33	0
Treatment	4	20	20	4	40	8	8	38	75	31
Treatment	5	20	15	3	35	7	0	43	100	24
Treatment	6	20	20	4	25	5	0	80	100	6
